# Comparative Analysis of In-House and Commercially Available Media for Determining the Antifungal Susceptibility Profile of Candida Species

**DOI:** 10.7759/cureus.76591

**Published:** 2024-12-29

**Authors:** Savita B Tajane, Satyajeet Pawar, Shivaji T Mohite, Satish R Patil

**Affiliations:** 1 Department of Microbiology, Krishna Institute of Medical Sciences, Krishna Vishwa Vidyapeeth (Deemed To Be University), Karad, IND

**Keywords:** antifungal susceptibility testing, c. albicans, candida species, disc diffusion, non-albicans candida spp

## Abstract

Background

The emergence of treatment-resistant *Candida* species has highlighted the importance of antifungal susceptibility testing as it is difficult to determine therapeutics solely based on species identification. However, as compared to bacterial pathogens, antimicrobial susceptibility testing in fungi still remains underutilized in most clinical diagnostic microbiological services. The disc diffusion (DD) technique is reported to be easy and cost-effective and therefore can be easily incorporated as a routine method. However, the selection of media remains the most crucial factor for antifungal susceptibility testing using the DD method. In the present study, in-house prepared and commercially available Mueller-Hinton agar with 2% glucose and 0.5 μg/ml methylene blue dye (MH-GMB) for determining the antifungal susceptibility profile of *Candida* species.

Method

The study involved 165 strains of eight different *Candida* species obtained from various clinical specimens. MH-GMB was the media used for antifungal susceptibility testing of *Candida* strains, and the efficacy of in-house and commercially available MH-GMB was compared.

Results

All *Candida* isolates showed sufficient growth on readymade MH-GMB procured from commercial sources. The frequency of trailing phenomenon was very low in MH-GMB procured from commercial sources. The visualization of zone margins was more enhanced with commercially procured MH-GMB compared to in-house media.

Conclusion

As per the available literature, the present study is the first to report the comparative account of in-house prepared and commercially procured MH-GMB for antifungal susceptibility testing of *Candida* spp. Commercially procured MH-GMB appears to be more advantageous over in-house prepared media as there is rapid growth, infrequent appearance trailing phenomenon, and more clear zone diameters. It is easy to weigh and prepare. Since it is pre-formulated, there is no chance of preparation error.

## Introduction

A while back, there was a dramatic upsurge in the incidence of fungal infection, especially candidiasis. Among the myriads of reasons responsible, the advent of HIV/AIDS, expanded exposure to wide-spectrum antimicrobials and immunosuppressants, long hospital stays, and indwelling medical devices are more important [1-2].

Candidiasis, though usually recognized as an opportunistic infection, is increasingly now noted as the primary cause of infection, particularly in hospitalized patients [3]. Although *C. albicans* is the most pervasive pathogenic species, recent studies have documented the emergence of non-albicans* Candida* (NAC) spp. The clinical manifestation of NAC spp. is similar to that of *C. albicans*; however, infections caused by NAC spp. are often difficult to treat, as NAC spp. demonstrate reduced susceptibility to antifungal drugs [4-5].

The significant increase in the isolation of treatment-resistant species has highlighted the importance of performing antifungal susceptibility testing, as determining therapeutic options solely on the basis of species identification is challenging [6]. However, as compared to antibacterial susceptibility testing, antifungal susceptibility testing remains underutilized in most clinical diagnostic microbiological services [7].

For *Candida *spp., the Clinical and Laboratory Standards Institute (CLSI) recommends two primary techniques for antifungal susceptibility testing: broth dilution (macro- and microdilution) and the disc diffusion (DD) method. The CLSI broth microdilution (BMD) method serves as the gold standard (reference) for antifungal susceptibility testing. However, BMD is labor intensive and often costly to be performed on a routine basis especially if isolates to be tested are less. On the other hand, the DD technique is reported to be easy and cost-effective and therefore can be easily incorporated as a routine method [8-9]. The trailing phenomenon often complicates the reading of antifungal susceptibility testing. It is due to the growth of residual organisms that occurs beyond the minimum inhibitory concentration (MIC) that causes difficulty in visualization and interpretation of results. It is extremely necessary to differentiate between true resistance and trailing growth for the effective implementation of the clinical utility of antifungal susceptibility testing.

As per CLSI, Mueller-Hinton agar with 2% glucose and 0.5 μg/ml methylene blue dye (MH-GMB) is the recommended medium for antifungal susceptibility testing of *Candida* spp. [9]. As the MH-GMB was not commercially available, the laboratories usually prepare this media in-house [10]. However recently, MH-GMB has been available from commercial sources. The present study aimed to compare the performance of in-house prepared and commercially procured (MH-GMB) for determining antifungal susceptibility testing (AFST) of *Candida* species. The objective was to compare growth patterns, zone clarity, and the occurrence of the trailing phenomenon on both media, as well as to evaluate the feasibility and advantages of using commercially available MH-GMB for routine antifungal susceptibility testing. This study is important because accurate antifungal susceptibility testing is crucial for guiding effective therapy, especially with the increasing emergence of treatment-resistant *Candida* species.

## Materials and methods

The study was conducted after obtaining approval from the Institutional Ethics Committee of Krishna Institute of Medical Sciences, Karad (approval no. KIMSDU/IEC/04/2023). Species identification was made based on the results of the germ tube test, colony color, and morphology on HiCrome™ Candida differential agar (HiMedia, Mumbai, India), whereas the VITEK® 2 Compact System (version 9.02, bioMérieux, France) was used for confirming the species. Specifically, *Candida auris* isolates were confirmed using MALDI-TOF-MS (matrix-assisted laser desorption ionization-time-of-flight mass spectrometry). In total, 165 strains, representing eight different species of *Candida* from clinical specimens, were part of the study. These included *C. albicans* (65), *C. tropicalis* (57), *C. parapsilosis *complex (11), *C. krusei *(9), *C. auris (*7), *C. kefyr* (7), *C. glabrata* (6), and *C. lusitaniae *(3).

Antifungal susceptibility testing

Antifungal susceptibility testing of these strains was performed by the DD technique as illustrated in the M44-A document of CLSI "Method for Antifungal Disk Diffusion Testing of Yeasts." Quality control was carried out using *C. parapsilosis* (American Type Culture Collection, ATCC 22019), *C. krusei *(American Type Culture Collection, ATCC 6258), and *C. albicans* (American Type Culture Collection, ATCC 90028). The results of the antifungal susceptibility tests were interpreted as per the standard CLSI guidelines.

Media

In this study, MH-GMB was the media used for antifungal susceptibility testing of *Candida* strains, and the efficacy of in-house and commercially available MH-GMB was compared. The in-house-MH-GMB was prepared by adding 2% glucose (pre-sterilized) and 0.0005 gm methylene blue to 1000 mL of pre-sterilized MH agar (HiMedia Laboratories Pvt. Ltd., Mumbai, India). The ready-made MH-GMB was obtained from HiMedia Laboratories Pvt. Ltd. (Mumbai, India).

Antifungal Drugs

*Candida* strains were tested against antifungal drugs, including itraconazole (10 µg), voriconazole (1 µg), and fluconazole (25 µg). The antifungal discs were obtained from HiMedia Laboratories Pvt. Ltd.

Inoculum Preparation

To obtain the desired inoculum, five distinct colonies of approximately 1 mm from a 24-hour-old culture grown on Sabouraud's dextrose agar (SDA) were picked. Approximately five to six colonies were picked and suspended in 5 ml of sterile 0.85% saline and vortexed. The resultant turbidity was adjusted using a spectrophotometer (530 nm wavelength) to yield 1 x 10^6 ^to 5 x 10^6^ cells/ml. The suspension was compared with the 0.5 McFarland standard.

Inoculation of Media

Approximately, 20 minutes after adjusting the inoculum isolated to be tested, a sterile cotton swab was dipped into the prepared inoculum suspension. This swab was then rotated many times and firmly pressed against the upper inner wall of the test tube containing inoculum suspension in order to express the excess fluid.

The MH-GMB (both in-house prepared and commercially procured) plates were dried for 15 minutes in a clean incubator meant for drying media before inoculation. The surface of such dried MH-GMB was then inoculated with the test inoculum evenly by swabbing the entire surface of the media. The process of inoculating the media was repeated thrice by turning the plate at a 60°C angle and after that, the rim of agar was inoculated. Both in-house and commercially procured MH-GMB media were simultaneously inoculated with the same inoculum.

Application of Antifungal Discs

After the inoculated strain was allowed to dry on the surface of the media, antifungal discs were aseptically dispensed onto the surface of the inoculated agar plates. Each disc was lightly pressed to ensure complete contact with the agar. The discs were spaced 24 mm apart on the agar. Finally, the inoculated plates were inverted and incubated within 15 minutes of the application of the discs.

Reading and interpretation of the results

Following incubation at 35(±2) °C for 24 hours, if insufficient growth was observed after 24 hours, the plates were read after 48 hours. The plate was held a few inches above a black, non-reflecting background illuminated with reflected light. The zone diameter was measured using a zone scale. The interpretive categories were susceptible (S), susceptible-dose dependent (SDD), intermediate (I), resistant (R), and non-susceptible (NS). *C. parapsilosis* (American Type Culture Collection, ATCC 22019), *C. krusei* (American Type Culture Collection, ATCC 6258), and *C. albicans* (American Type Culture Collection, ATCC 90028) were used as quality control strains [9].

Statistical analysis

The zone diameters of each isolate on in-house prepared and commercially procured MH-GMB media were entered in Microsoft Excel (Microsoft Corporation, USA). The data were analyzed using IBM SPSS Statistics for Windows, Version 28.0 (released 2021, IBM Corp., Armonk, NY). The mean (± SD) of zone diameters on both media were compared using the Mann-Whitney U test. A p-value of <0.05 was considered statistically significant.

## Results

Out of 165 *Candida *spp. included in the study, a total of 109 (66.06%) isolates showed the growth of in-house prepared MH-GMB, whereas 56 (33.93%) isolates showed sufficient growth after 48 hours of incubation. All isolates of *C. albicans *showed growth on in-house MH-GMB after 24 hours, whereas all isolates of *C. auris *and *C. glabrata* required 48 hours of incubation to produce sufficient growth. On the contrary, all *Candida* isolates showed sufficient growth in commercial MH-GMB media. The trailing phenomenon was noted in a total of 31 (18.78%) isolates on in-house prepared MH-GMB media, while the frequency of trailing phenomenon was very low in MH-GMB procured from commercial sources. It was noted only in two (1.21%) isolates.

**Figure 1:**
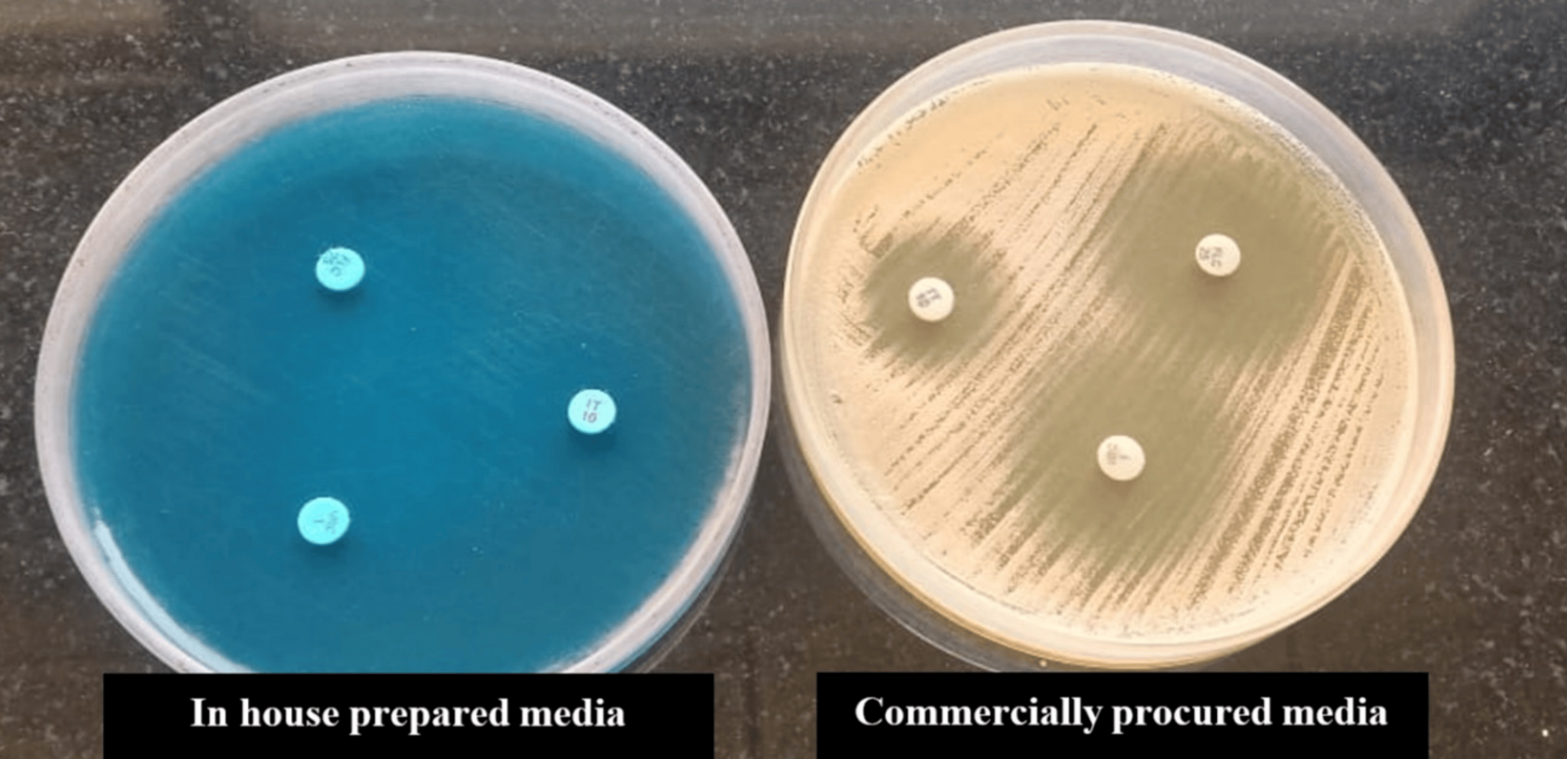
Zone margins on the in-house prepared and commercially procured Mueller-Hinton agar with 2% glucose and 0.5 μg/ml methylene blue dye (MH-GMB)

As shown in Figure 1, the visualization of zone margins was more enhanced with commercially procured MH-GMB compared to in-house media. Similarly, the occurrence of microcolonies near the center of the inhibition zone was infrequent with commercially procured MH-GMB.

As shown in Table 1, when the size of the zone of inhibition of *Candida* spp. on in-house and commercially procured MH-GMB were compared, there was no significant difference noted between the sizes of the zones of inhibition produced by *Candida *spp. on these media (Mann-Whitney U test <0.05). By the DD method, all seven isolates of *C. auris *were resistant to fluconazole, voriconazole, and itraconazole. Among 57 isolates of *C. tropicalis*, two (3.5%) showed resistance to fluconazole. In addition, one (16.7%) of the six *C. glabrata* isolates showed resistance to fluconazole. No strain of *C. albicans* was resistant to antifungal drugs. Antifungal resistance was not in NAC spp. like *C. parapsilosis,* *C. kefyr*, and *C. lusitaniae*.

**Table 1:** Comparison between the in-house and commercially available media for antifungal susceptibility testing of Candida isolates N: number, IH: in-house prepared media, CA: commercially available media, S (%): susceptible percentage, I (%): intermediate percentage, R (%): resistant percentage, *C. albicans*: *Candida albicans*, *C. tropicalis*: *Candida tropicalis*, *Candida parapsilosis* complex: *Candida parapsilosis*, *C. krusei*: *Candida krusei*, *C. auris*: *Candida auris*, *C*. *kefyr*: *Candida kefyr*, *C. glabrata*: *Candida glabrata*, *C. lusitaniae*: *Candida lusitaniae*, Mean (± SD): mean zone size in millimeters with standard deviation, statistical test: Mann-Whitney U Test used to compare the mean zone sizes between the in-house prepared and commercially available media. A value of p < 0.05 was considered statistically significant.

Candida isolate (N)	Media	Fluconazole				Itraconazole				Voriconazole			
		Mean zone size (± SD) in mm	S (%)	I (%)	R (%)	Mean zone size (±) SD in mm	S (%)	I (%)	R (%)	Mean zone size (±) SD in mm	S (%)	I (%)	R (%)
*C. albicans* (65)	IH	26.8±2.3	65 (100)	-	-	21.6±1.4	65 (100)	-	-	28.1±0.8	65 (100)	-	-
	CA	27.6±1.8	65 (100)	-	-	22.1±2.0	65 (100)	-	-	27.4±1.2	65 (100)	-	-
*C. tropicalis* (57)	IH	20.6±0.4	55 (96.5)	-	02 (3.5)	21.8±0.2	57 (100)	-	-	30.2±0.4	57 (100)	-	-
	CA	21.6±0.2	55 (96.5)	-	02 (3.5)	22.1±0.5	57 (100)	-	-	29.8±0.5	57 (100)	-	-
*C. parapsilosis *complex (11)	IH	22.2±0.6	11 (100)	-	-	24.5±0.5	11 (100)	-	-	26.2±0.8	11 (100)	-	-
	CA	22.8±0.8	11 (100)	-	-	25.2±0.4	11 (100)	-	-	25.8±0.4	11 (100)	-	-
*C. krusei *(9)	IH	Not tested (Innately resistant)				23.6±0.9	09 (100)	-	-	25.2±0.2	09 (100)	-	-
	CA					24.1±0.6	09 (100)	-	-	26.2±0.4	09 (100)	-	-
*C. auris* (7)	IH	12.2±1.8	-	-	07 (100)	11..5±0.5	-	-	07 (100)	14.2±0.2	-	-	07 (100)
	CA	12.8±1.2	-	-	07 (100)	11.9±0.2	-	-	07 (100)	14.8±0.4	-	-	07 (100)
*C. kefyr *(7)	IH	27.8±0.4	07 (100)	-	-	28.2±0.2	07 (100)	-	-	29.2±0.2	07 (100)	-	-
	CA	28.1±0.2	07 (100)	-	-	29.2±0.1	07 (100)	-	-	29.4±0.4	07 (100)	-	-
*C. glabrata *(6)	IH	22.2±0.5	05 (83.3)	-	01 (16.7)	25.7±1	06 (100)	-	-	24.8±1.2	06 (100)	-	-
	CA	22.4±0.4	05 (83.3)	-	01 (16.7)	26.2±0.8	06 (100)	-	-	25.1±0.9	06 (100)	-	-
*C. lusitaniae *(3)	IH	24.7±1	03 (100)	-	-	26.1±1.2	03 (100)	-	-	30.1±1.2	03 (100)	-	-
	CA	25.1±0.8	03 (100)	-	-	28.2 ±1.8	03 (100)	-	-	30.8±1.4	03 (100)	-	-

## Discussion

*Candida *spp., generally regarded as a part of normal commensal flora of humans, especially of the gastrointestinal and genital tract, were previously deemed as clinically insignificant or opportunists with minimal pathogenicity [6].

However, extensive research studies over the last few decades have supported the fact that *Candida *spp. is a potentially important cause of a broad range of illnesses, especially healthcare-associated infections, and is clinically relevant in terminally ill patients requiring admission to ICU and those having indwelling medical devices and on broad-spectrum antibiotics [6]. Limited antifungal drugs and the emergence of more resistance species further unnerves the treatment [6].

The literature states that in contrast to bacterial pathogens, acquired (secondary) resistance, though noted, is rare in fungi including *Candida*. With candidiasis being a non-contagious infection, there is almost no or minimal chance of patient-to-patient transfer of treatment-resistant strains. Similarly, resistance in *Candida* isolates is not plasmid-mediated, and therefore it is due to exposure to an antifungal therapy [11-12].

The abrupt surge in the incidence of infections due to NAC species has changed the global scenario of the antifungal resistance pattern of *Candida* isolates. The antifungal susceptibility pattern of NAC spp. cannot be predicted solely based on species identification, as NAC spp. exhibits a disparity in the degree of resistance to frequently used prophylactic and therapeutic antifungal drugs [6].

Emphasizing the significance of antifungal susceptibility testing of *Candida* spp, the guidelines of the Infectious Diseases Society of America (IDSA) mentioned that antifungal susceptibility testing is crucial for both the pervasive *C. albicans* and the cryptic emergent NAC spp. In the case of *C. albicans*, susceptibility testing is crucial to guide therapy, particularly in patients with persistent candidemia or other types of invasive candidiasis, while in NAC spp., it is of prime importance especially in patients who have prophylactic or therapeutic received dose of azoles [13].

Although in vitro antifungal susceptibility testing is now standardized and routinely utilized in developed countries, its utility in developing countries is still too limited due to its complexity, high technical demand, and high cost [7]. Compared to BMD, the DD method is more appealing due to its simplicity and cost-effectiveness. This method of antifungal susceptibility is the same as the Kirby-Bauer method, which is routinely utilized for antibacterial susceptibility testing [14]. The selection of media is the most crucial factor for antifungal susceptibility testing using the disk diffusion method (dDD). Yeast nitrogen base glucose (YNBG), antibiotic medium 3, Rosewell Parker Memorial Institute (RPMI) agar with 2% glucose (RPG), Casitone, and un-supplemented Mueller Hinton agar were used by several researchers for determination of antifungal susceptibility of *Candida* spp. [10]. As per the CLSI guidelines, MH-GMB is the recommended media for antifungal susceptibility testing by the DD method. MH-GMB supports the growth of all medically important *Candida *spp.

Barry et al. were the first to utilize MH-GMB for determining the antifungal susceptibility profile of *Candida* isolates [15]. Thereafter, MH-GMB has been also used by several workers and reported to be cost-effective, simple, and accurate media for determining antifungal susceptibility of *Candida *spp. [16]. Methylene blue in these mediums aids in improving the zone diameter determination/reading. Glucose augments the growth of *Candida* spp. [10].

For many years, there was no commercial source of MH-GMB. MH agar was either both prepared and supplemented with glucose and methylene blue in the laboratory or alternately the surface of commercially available MH agar plates was flooded with glucose and methylene blue solution [10]. When methylene blue and glucose solution is added to the surface of the MH agar, the plates should be air-dried before inoculation. Both prepared and flooded plates should be used within 24 hours of preparation [10]. However, recently MH-GMB has been available commercially. Several researchers utilize this medium for performing antifungal susceptibility testing on *Candida* isolates. Debta et al. (2024) found that *Candida* isolates exhibited minimal resistance to itraconazole and amphotericin B, with *C. tropicalis* showing 60% resistance to fluconazole [17]. By contrast, Ali et al. (2024) found that *C. albicans* was highly susceptible to amphotericin B (92.4%) and ketoconazole (91.7%), while fluconazole resistance was noted in *C. tropicalis *and *C. glabrata*. Ketoconazole had the highest sensitivity (89.7%) [18]. However, there is no comparative study of both in-house prepared and commercially procured media for antifungal susceptibility testing. This study was conducted to assess whether there is any difference in the efficacy of in-house prepared and commercially procured media, a comparison that, to our knowledge, has not been documented in the literature.

In the present study, when the efficacy of in-house prepared and commercially procured MH-GMB media was compared by evaluating the mean of zone size and applying the Mann-Whitney U test, it was noted that though the interpretative criteria with both (in-house and commercially prepared) media were the same, commercially procured MH-GMB had several advantageous over in-house prepared media. All *Candida *spp. showed sufficient growth after 24 hours of incubation on commercially procured media, whereas on in-house prepared, 33.93% of isolates showed sufficient growth only after 48 hours. The trailing phenomenon was infrequent with the use of commercially procured MH-GMB. The trailing phenomenon often complicates the interpretation of results especially azoles, where reduced but persistent *Candida* growth in high concentrations confuses end-point determination [19]. The trailing phenomenon may vary as per media, the incubation temperature, and other variables of media like pH and glucose concentration [19]. Moreover, the appearance of microcolonies near the center of the inhibition zone was not seen in commercially procured MH-GMB. Based on these features, the commercially procured media appears to be more effective for the utilization of antifungal susceptibility testing of *Candida* isolates. The CLSI guidelines are not yet established for all antifungals for *Candida* isolates. Moreover, the interpretation of the trailing phenomenon is subjective causing limitations for the generalization of the findings.

## Conclusions

As per the available literature, the present study is the first to report the comparative account of in-house prepared and commercially procured MH-GMB for antifungal susceptibility testing of *Candida *spp. In conclusion, commercially procured MH-GMB appears to be more advantageous over in-house prepared media as there is rapid growth, infrequent appearance trailing phenomenon, and clearer zone diameters. It is easy to weigh and prepare. Since it is pre-formulated, there are no chances of preparation error.
